# Prenatal Diagnosis of Autosomal Recessive Renal Tubular Dysgenesis with Anhydramnios Caused by a Mutation in the *AGT* Gene

**DOI:** 10.3390/diagnostics9040185

**Published:** 2019-11-11

**Authors:** Gwo-Chin Ma, Ying-Chung Chen, Wan-Ju Wu, Shun-Ping Chang, Ting-Yu Chang, Wen-Hsiang Lin, Ming Chen

**Affiliations:** 1 Department of Genomic Medicine and Center for Medical Genetics, Changhua Christian Hospital, Changhua 50046, Taiwan; 128729@cch.org.tw (G.-C.M.); 176531@cch.org.tw (Y.-C.C.); crystalwu835@gmail.com (W.-J.W.); 70914@cch.org.tw (S.-P.C.); taiwanbird@gmail.com (T.-Y.C.); 397620cch@gmail.com (W.-H.L.); 2 Department of Genomic Science and Technology, Changhua Christian Hospital Healthcare System, Changhua 50046, Taiwan; 3 Department of Medical Research, Changhua Christian Hospital, Changhua 50006, Taiwan; 4 Department of Biomedical Engineering, Chung Yuan Christian University, Taoyuan 32023, Taiwan; 5 Department of Medical Laboratory Science and Biotechnology, Central Taiwan University of Science and Technology, Taichung 40601, Taiwan; 6 Department of Obstetrics and Gynecology, Changhua Christian Hospital, Changhua 50006, Taiwan; 7 Department of Biomedical Science, Dayeh University, Changhua 51591, Taiwan; 8 Department of Obstetrics and Gynecology, College of Medicine, National Taiwan University, Taipei 10041, Taiwan; 9 Department of Medical Genetics, National Taiwan University Hospital, Taipei 10041, Taiwan; 10 Department of Life Science, Tunghai University, Taichung 40704, Taiwan

**Keywords:** ARRTD, oligohydramnios, anhydramnios, MCA-REDF, autosomal recessive, renin-angiotensin system, prenatal diagnosis

## Abstract

Autosomal recessive renal tubular dysgenesis (ARRTD) is a rare and lethal disorder that causes stillbirth or early neonatal death. Most of the reported cases are diagnosed postnatally by a histopathological hallmark of the absence or paucity of differentiated proximal tubules in kidneys. Prenatal diagnosis of ARRTD is challenging because only a few fetal features (e.g., oligohydramnios/anhydramnios, anuria) are associated with this condition. In this study, we report a fetus with ARRTD, which showed anhydramnios and invisible urinary bladder since the second trimester, followed by growth restriction and reversed end diastolic flow in the middle cerebral artery (MCA-REDF). No morphological anomaly was detected on the fetal kidneys during an ultrasound scan. The baby died of refractory hypotension the day after their birth. Genetic analysis of genes that are involved in the renin-angiotensin-aldosterone system (RAAS), which are the known genetic causes of ARRTD, identified a novel, biparental-origin homozygous c.857-619_1269+243delinsTTGCCTTGC mutation in the *AGT* gene. The mutation is considered as pathogenic because it is cosegregated with ARRTD and detected in other unrelated ARRTD families. Our findings link the fetal ultrasound manifestations to the ARRTD, highlighting clues that are useful for prenatal diagnosis, which warrants confirmatory genotyping of the RAAS genes including oligohydramnios/anhydramnios, anuria (absent filling of a fetal urinary bladder), MCA-REDF, and a morphologically normal kidney.

## 1. Introduction

Renal tubular dysgenesis (RTD; OMIM #267430) is a rare and lethal disorder that affects renal development before birth and causes intrauterine fetal demise or early neonatal death. Most of the reported cases are diagnosed postnatally based on a histopathological hallmark of the absence or paucity of differentiated proximal tubules in kidneys [1]. Additional features that are observed in affected births include refractory hypotension and poor ossification of the skull, resulting in wide cranial sutures and large fontanels [2,3,4]. Prenatal diagnosis of RTD is challenging because only a few fetal features have been linked to this condition. Oligohydramnios/anhydramnios is the most common sign that is noted in fetuses with RTD [2,5]. Deficiency of amniotic fluid causes fetal compression and growth restriction that may lead to Potter sequence, such as facial dysmorphism, limbs positioning defects, arthrogryposis, and lung hypoplasia [6]. Anuria is another fetal sign that is commonly seen in RTD [1,2,5]. Fetal urine is the major constituent of amniotic fluid. Early onset and persistent fetal anuria leads to oligohydramnios/anhydramnios and the Potter sequence [6,7].

RTD can be acquired during fetal development due to drugs or certain disorders [8,9,10,11,12] or can be inherited in an autosomal recessive manner as so called autosomal recessive RTD (ARRTD). ARRTD is caused by mutations in genes that are involved in the renin–angiotensin–aldosterone system (RAAS), including *AGT* (angiotensinogen; OMIM +106150), *REN* (renin; OMIM *179820), *ACE* (angiotensin-converting enzyme; OMIM +106180), and *AGTR1* (angiotensin II receptor type 1; OMIM *106165) [3,13,14]. RAAS proteins are involved in a series of steps to produce angiotensin II protein, which is responsible for regulating blood pressure, fluid and electrolyte balance, as well as systemic vascular resistance [15]. RAAS is essential during human fetal development and dysfunction of the RAAS has been implicated as a cause of persistent low blood pressure, which may inevitably affect the skull membrane bone which is highly vascular and requires high oxygen tension for normal ossification [16]. Mutation screening of RAAS genes has been an acceptable approach for the genetic diagnosis of ARRTD [13,14].

Further, ARRTD poses a dilemma for families and physicians during prenatal genetic counseling because almost all reported cases have resulted in fatal outcomes. Early recognition of fetal ARRTD on the basis of clinical and ultrasound analyses and the characterization of the genetic defects permits genetic counseling and early prenatal diagnosis. We report here the results of a clinical and genetic study of a prenatal case with ARRTD. We highlight the clues that may be useful for prenatal diagnosis and report a specific homozygous *AGT* mutation that is associated with ARRTD.

## 2. Case Report

**Figure 1:**
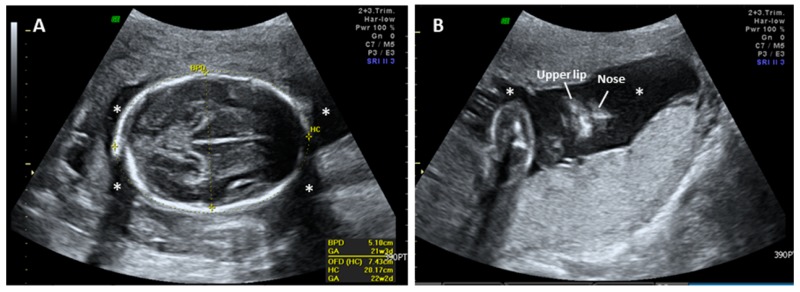
Prenatal ultrasound images of the fetus with renal tubular dysgenesis (RTD) at 22^+2^ weeks of gestational age (wGA) showing structural normality without oligohydramnios. (**A**) Axial and (**B**) coronal view of the fetus. The black space surrounding the fetus is amniotic fluid (stars). BPD: biparietal diameter, HC: head circumference.

**Figure 2:**
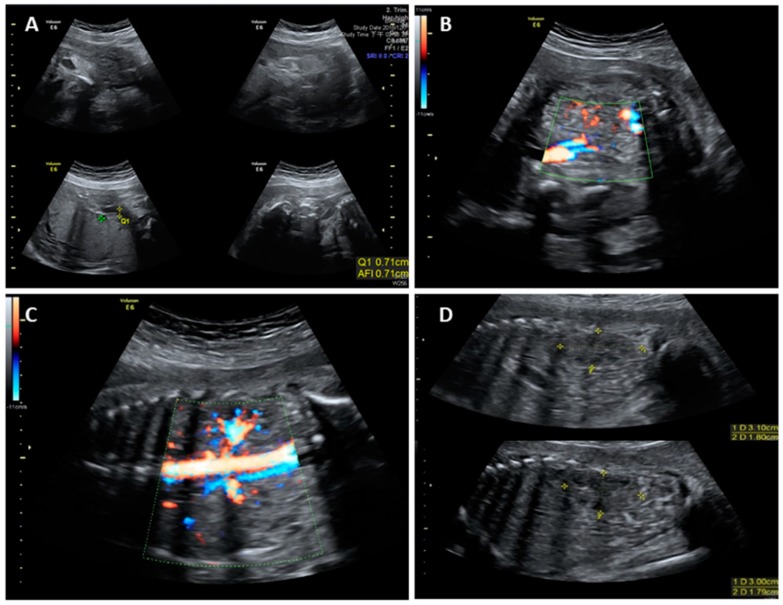
Prenatal ultrasound images of the fetus with RTD at 28^+6^ wGA showing (**A**) severe oligohydramnios (amniotic fluid index, AFI = 0.71), (**B**) invisible bladder, (**C**) visible bilateral renal arteries, and (**D**) morphologically normal kidneys with a proper size and corticomedullary differentiation (upper: right kidney; lower: left kidney).

This work did not form part of a research project, but is rather a retrospective case report. Neither ethical approval nor informed consent is necessary for publication. A 32-year-old Taiwanese woman, gravida 2 para 1, was referred to our hospital at 28^+6^ weeks gestational age (wGA) because of unexplained severe oligohydramnios. Medical records showed that the pregnant woman received level II ultrasonographic screening at 22^+2^ wGA and the fetus was structurally normal and surrounded with amniotic fluid (Figure 1). However, at 26 wGA, severe oligohydramnios was noted. Meanwhile, steroid administration was provided to promote lung maturation. The woman had no medical underlying diseases such as diabetes mellitus, hypertension, thrombophilias, and renal diseases and had no obstetric conditions such as preeclampsia that may be associated with uteroplacental insufficiency. She also denied consanguineous marriage and any remarkable surgical history. During her visit at 28^+6^ wGA, a nitrazine test for membrane ruptures was performed and the result was negative. The values of maternal serum antiphospholipid antibodies were all within the normal ranges. Follow-up ultrasonographic exams revealed an anatomically normal fetus with an appropriate estimated fetal weight. However, severe oligohydramnios (amniotic fluid index, AFI = 0.71) and an invisible bladder were noted (Figure 2A,B). The renal scans were normal, including visible bilateral renal arteries, proper size of biometry [17], and fair corticomedullary differentiation (Figure 2C,D).

The pregnant woman consented to a placental biopsy at 30 wGA for cytogenetic analysis and array-based comparative genomic hybridization (aCGH). Both tests showed that the fetus had a normal male karyotype 46,XY and genomic composition arr(1–22)x2,(X,Y)x1. After nondirective counselling, she continued pregnancy and regular antenatal care. Close surveillance of the pregnancy was advised.

**Figure 3:**
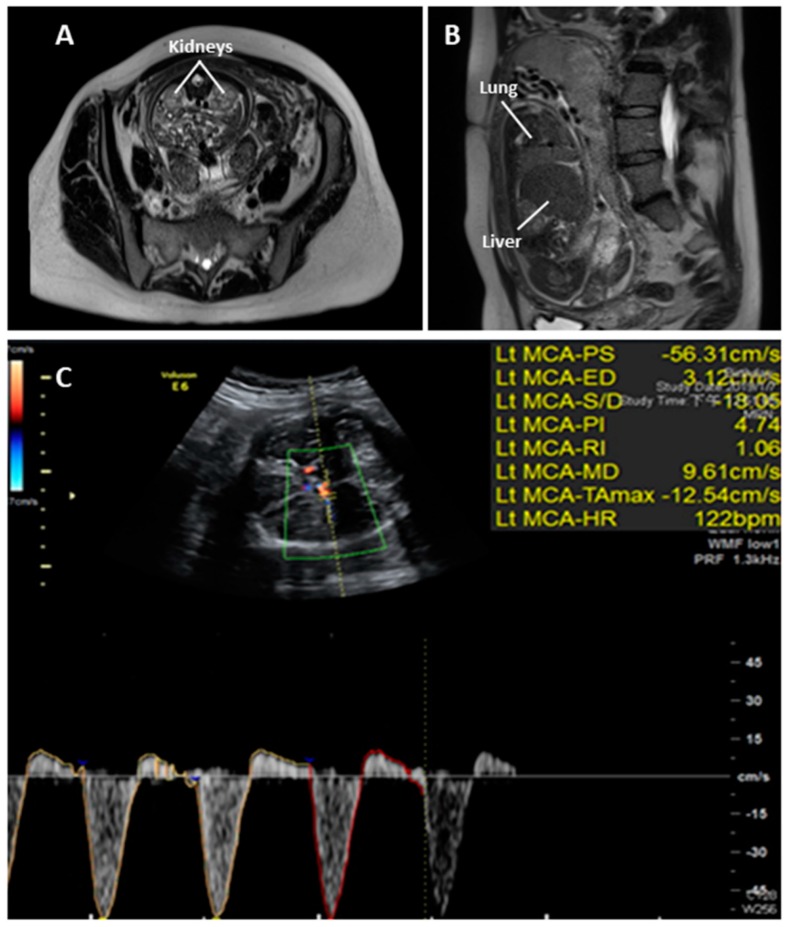
Magnetic resonance imaging (MRI) T2-weighted sagittal view of the fetus with RTD at 30^+5^ wGA showing (**A**) bilateral anatomically normal kidneys and (**B**) decreased signal intensity of the lungs, suggesting poor fetal lung maturation. Prenatal ultrasonography at 33^+2^ wGA showing (**C**) reversed end diastolic flow in the middle cerebral artery (MCA-REDF).

Fetal magnetic resonance imaging (MRI) for kidney and lung evaluation at 30^+5^ wGA revealed bilateral anatomically normal kidneys and decreased signal intensity of her lungs and invisible bladder (Figure 3A,B). Fetal ultrasonography was subsequently performed at 33^+2^ wGA: anhydramnios, invisible urinary bladder, small fetal size for GA (estimated fetal weight was 1733 gm, which is between the 5th and 10th percentile), and reversed end diastolic flow in the middle cerebral artery (MCA-REDF) were noted (Figure 3C).

The pregnant woman was admitted at 33^+4^ wGA because of antepartum hemorrhage. A second course of steroid administration was then provided and she underwent an emergent cesarean section at 33^+5^ wGA because of fetal distress. After birth, the Apgar score of the first and fifth minutes were 5 and 6, respectively. Poor spontaneous breathing with a low heart rate (<60 bpm) was noted. An endotracheal tube was then inserted and the heart rate of the baby recovered to 160 to 170 bpm. After the baby was transported to NICU, the first recorded blood pressure was 28/18 mmHg and an echocardiogram was then arranged and revealed pulmonary arterial hypertension (pulmonary artery systolic pressure was around 40 to 45 mmHg). Right side pneumothorax was noted by a chest X-ray. Despite inotropic agents and inhaled nitric oxide (iNO) treatment, the baby expired 22 h after birth.

**Figure 4:**
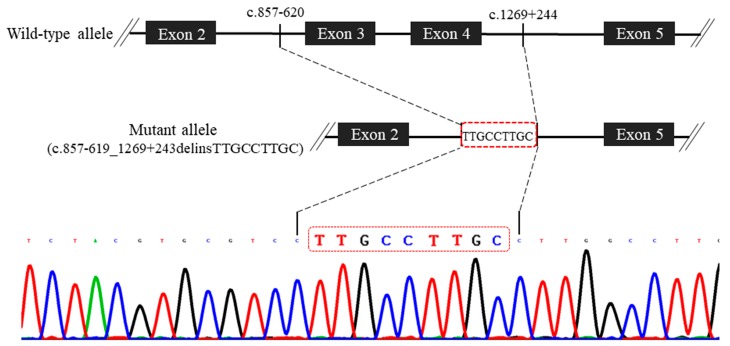
Schematic diagram of the novel c.857-619_1269+243delinsTTGCCTTGC mutation in the *AGT* gene. The mutation is caused by a deletion of nucleotides c.857-619 to c.1269+243, replaced by nucleotides TTGCCTTGC.

The couple declined an autopsy but accepted further molecular investigation by sampling of the placenta. Mutation screening in all coding exons and their corresponding flanking intron sequences of four RAAS genes (*AGT*, *REN*, *ACE*, and *AGTR1*) was carried out by polymerase chain reaction (PCR) and direct sequencing. Initially, no sequence variant was directly recognized. However, repeated PCR failures of exon 3 and exon 4 of the *AGT* gene suggested gross deletion(s) involving these two exons. Long range PCR was subsequently performed and a homozygous c.857-619_1269+243delinsTTGCCTTGC mutation was identified (Figure 4). The mutation contained a 2870-base pair (bp) genomic deletion of nucleotides c.857-619 to c.1269+243, replaced by nucleotides TTGCCTTGC, leading to a truncated protein or removal of the mutant mRNA by nonsense-mediated RNA decay [18], which is absent from The Human Gene Mutation Database (available online: http://www.hgmd.cf.ac.uk/ac/index.php, 15 October 2019). This mutation was found in the heterozygous status in the unaffected mother and father, pointing to the biparental origin of the described mutation and further supporting a fetal case of ARRTD. To improve the diagnosis, an in-house designed PCR was provided for rapid detection of this specific c.857-619_1269+243delinsTTGCCTTGC mutation in the *AGT* gene (Supplementary Material: Figure S1). In short, a triple primer set was used for differentially amplifying the wild type allele and the mutant allele (with the *AGT* mutation). The wild type allele was expected to yield a 343-bp amplicon and the mutant allele will yield a 250-bp amplicon under a definite PCR condition (Supplementary Material: Figure S1).

## 3. Discussion

Diagnosis of ARRTD is challenging and is usually established by histology. However, renal biopsy for histological analysis is not feasible for all cases, especially in fetuses. Molecular genetic analysis provides an alternative method which enables more accurate diagnosis of this disease, given that ARRTD has a well-characterized genetic basis. This study described a prenatal ARRTD case that was diagnosed based on fetal ultrasound findings and mutation screening of the RAAS genes.

As is known, the RTD phenotype may be acquired, which is originated from in utero non-genetic conditions leading to renal hypoperfusion, such as twin-to-twin transfusion syndrome [8,9], renal stenosis [8,9], and exposure to RAAS blockers (e.g., angiotensin-converting enzyme inhibitor [10] or AT1 antagonist [11,12]). The prevalence of ARRTD remains unknown. According to an autopsy study on fetuses and neonates, only 7 (58%) of the 12 detected RTD cases were recognized as ARRTD [19]. As a result, prenatal diagnosis of ARRTD should be clinically based on anamnesis, ultrasonography, and exclusion of other secondary origins [14].

For the prenatal sonographic findings, only a few reports mentioned the features of ARRTD because the disease was probably underdiagnosed [13,14]. Oligohydramnios/anhydramnios is the most common and possibly the only sign of ARRTD in the prenatal period, which may be recognized from variable gestational age during the second trimester [15]. Most fetuses with ARRTD presented oligohydramnios/anhydramnios at 20 to 22 wGA [2], however cases with late onset of oligohydramnios/anhydramnios after 22 wGA (even until 27 to 28 wGA) were reported [20]. In our case, the amniotic fluid was adequate at 22 wGA and severe oligohydramnios was noted at 26 wGA. As a result, a delayed onset of severe oligohydramnios between 22 to 26 wGA was suggested, which could be missed in regular anatomic screening between 18 and 22 wGA. Poor fetal lung maturation was implicated for this case by magnetic resonance imaging (MRI) despite the fact that lung hypoplasia is rare in cases of oligohydramnios that occur after 25 weeks [21,22]. We thus considered that a fetal lung maturation survey with ultrasonography/MRI should be arranged if the actual onset time of oligohydramnios/anhydramnios is unclear. An unexpected finding was that the fetus presented with MCA-REDF before birth. MCA-REDF is an extremely rare prenatal sonographic finding and only a few cases that were associated with fetal growth restriction, anemia, intraventricular hemorrhage, and a rare hepatic anomaly were reported in the literature; all documented cases suffered from poor neonatal outcomes [23]. We measured MCA Doppler at 33^+2^ wGA for this case because of small fetal size for GA and anhydramnios. However, we had no opportunity to follow the MCA Doppler result to figure out the whole picture of MCA-REDF in this case because the woman delivered the baby a few days after. It is possible that the MCA-REDF is as a result of increased fetal intracranial pressure caused by external compression of the fetal head. We proposed that if oligohydramnios/anhydramnios was prenatally noted but (1) the pregnant woman had no premature preterm rapture of the membrane, (2) no evidence of uteroplacental insufficiency, and (3) a normal fetal renal scan (including normal morphology with renal arteries, normal echogenicity, and no sign of obstructive uropathy, multicystic kidneys, and renal agenesis), then ARRTD should be enlisted in a differential diagnosis.

Four RAAS genes (*AGT*, *REN*, *ACE*, and *AGTR1*) have been reported to cause RAAS dysfunction, leading to ARRTD [13,14]. In 2012, Gribouval et al. [14] reviewed the mutation spectrum among 48 unrelated families with ARRTD, of which 31 (64.6%), 10 (20.8%), 4 (8.3%), and 3 (6.3%) families were affected by mutations in *ACE*, *REN*, *AGT*, and *AGTR1*, respectively. *AGT* mutations are relatively rare and so far, only 27 *AGT* mutations have been recorded in The Human Gene Mutation Database (http://www.hgmd.cf.ac.uk/ac/index.php). These mutations vary from missense, nonsense, splicing, and regulatory to small deletion. The c.857-619_1269+243delinsTTGCCTTGC mutation that we identified in the *AGT* gene is novel and is the first gross deletion that is associated with ARRTD. The deletion spanning across exon 3 and exon 4 of the *AGT* gene (totally, containing 5 exons) resulted in partial loss of the Serpin domain of the matured AGT protein [15]. This mutation was cosegregated with ARRTD in the fetus’ family. The two parents of the affected fetus were presumably unrelated (or distantly related). Furthermore, a mutant allele including the specific c.857-619_1269+243delinsTTGCCTTGC mutation in the *AGT* gene was also found in additional unrelated families of ARRTD in the Taiwanese population (personal communication with Dr. Min-Hua Tseng, his manuscript is under submission). This suggests that the homozygosity of the specific *AGT* mutation causes ARRTD and this specific allele may represent a result of the founder effect (i.e., the mutation originated from a small population of ancestors) or a combination of the founder effect and consanguinity. Further large-scale genetic studies may facilitate our understanding of ARRTD in the Taiwanese population. We provided an in-house designed PCR, allowing the rapid detection of this specific *AGT* mutation (Supplementary Material: Figure S1).

## 4. Conclusions

We reported this rare case by linking the prenatal ultrasound findings to the ARRTD, followed by confirmatory genotyping of the genes that are involved in the RAAS. ARRTD is rare and lethal and should be considered in fetuses with oligohydramnios/anhydramnios, anuria (absence filling of the fetal urinary bladder), and MCA-REDF without sonographic renal abnormality. Molecular genetic analysis can be used for rapid diagnosis of ARRTD when renal biopsy is not feasible. As was shown in this study, a novel homozygous *AGT* mutation as the underlying cause of ARRTD was detected in a fetus from a presumably nonconsanguineous, healthy Taiwanese couple, suggesting a likely founder effect or a combination of the founder effect and consanguinity. We believe that our results will significantly contribute to prenatal diagnosis and genetic counseling.

## Supplementary Materials

Supplementary materials can be found at https://www.mdpi.com/2075-4418/9/4/185/s1. Figure S1: Schematic diagram of the polymerase chain reaction (PCR) for rapid detection of the novel c.857-619_1269+243delinsTTGCCTTGC mutation in the *AGT* gene, which is presumably a hotspot mutation in the Taiwanese RTD population.
