# Supplementary material for: Prenatal Diagnosis of Autosomal Recessive Renal Tubular Dysgenesis with Anhydramnios Caused by a Mutation in the AGT Gene
Source: Diagnostics (Basel). 2019 Nov 11;9(4):185. doi: 10.3390/diagnostics9040185 (PMC6963964; doi:10.3390/diagnostics9040185)
Supplement: Supplementary file 1 [file diagnostics-09-00185-s001.pdf]

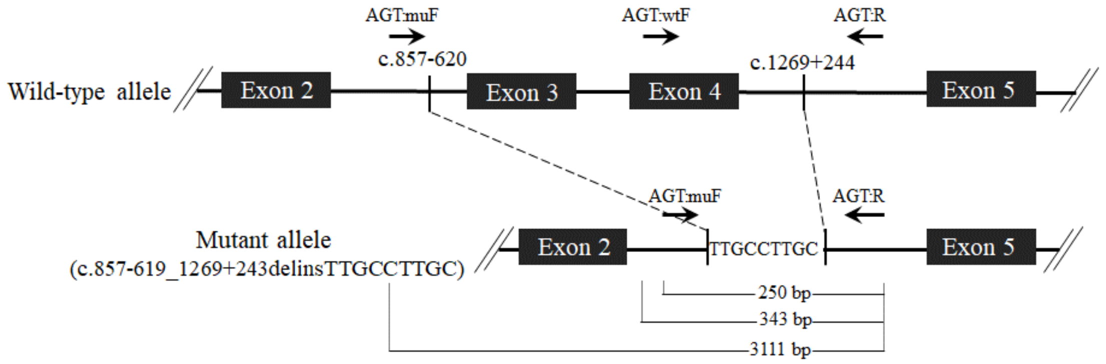

Figure S1. Schematic diagram of the polymerase chain reaction (PCR) for rapid detection of the novel c.857-619_1269+243delinsTTGCCTTGC mutation in *AGT* presumably a hotspot mutation in Taiwanese RTD population. Two different forward primers (AGT:muF and AGT:wtF) and one common reverse primer (AGT:R) were designed and used for PCR. The wild type allele yielded a 343-bp amplicon by the primer pair of AGT:wtF and AGT:R (notably, the wild type allele yielded no PCR product by the primer pair of AGT:muF and AGT:R because the amplicon size is too large to be amplified under the PCR condition we used), and the mutant allele (with c.857-619_1269+243delinsTTGCCTTGC mutation) yielded a 250-bp amplicon by primer pair of AGT:muF and AGT:R. Arrows indicated the primers. The primer sequences of the AGT:muF, AGT:wtF and AGT:R are 5’-CGTGGCCCACTCTGTATTCT-3’, 5’-AATTGAGCAATGACCGCATC-3’ and 5’-CTGTCTTCATCCCGGTTTCA-3’ respectively. PCR was performed with condition of 95 ℃, 5 min → (95 ℃, 40 sec → 55 ℃, 40 sec → 71 ℃, 1 min)$_{40}$→ 71 ℃, 1 min→4 ℃, 1 min.
